# Supplementary material for: Riemannian classification of single-trial surface EEG and sources during checkerboard and navigational images in humans
Source: PLoS One. 2022 Jan 14;17(1):e0262417. doi: 10.1371/journal.pone.0262417 (PMC8759639; doi:10.1371/journal.pone.0262417)
Supplement: S1 File — (DOCX) [file pone.0262417.s001.docx]

# Supplementary material

## Dataset and source files

The anonymized dataset necessary to replicate the study’s findings reported in this article, was made publicly available without restriction at [1]. The dataset consists of covariance matrices of shape $12\times12$, estimated on the output of the xDAWN filtering algorithm applied to either EEG signals or mean cortical sources activations of each trial of each subject.

The Python source files implementing the classification pipelines and generating the figures published in this article were also made publicly available without restriction at <https://github.com/cedricsimar/TC-EEG-RSTA>.

## Grey screens

During EEG recordings, in order to prevent the preceding stimulus from influencing the consecutive one, and thus potentially bias subsequent analyses, the presentation of each visual item was immediately followed by the presentation of a uniform grey image which resets the participant mental state before the presentation of the next visual item. In this context, the presented grey image is usually considered neutral, of small interest, and not carrying discriminative information. In this supplementary material, we performed a comparative ERP and RSTA analysis on the grey images in order to show that these images, typically considered neutral, actually carry discriminative information about the preceding visual stimulus presented to the subject.

Figure 10 illustrates the grand average ERP components (P100, N140, and P220) observed between the presentation of all visual stimuli, i.e. 3D-Tunnels, Checkerboards, grey images following a Tunnel, and grey images following a Checkerboard. Compared to the presentation of a 3D-Tunnel or a Checkerboard, the EEG signals of grey images presented a P100 and a N140 of smaller amplitude that were not followed by a P220.


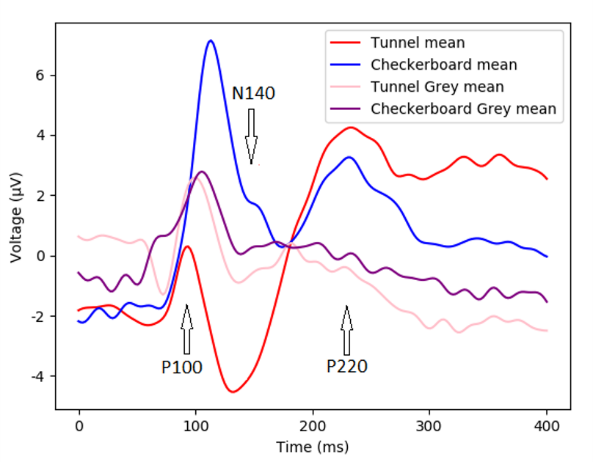


**Figure 10**. *Grand averaged* *ERP recorded on the occipital area (electrode Oz) corresponding to the 4 visual conditions: the Checkerboard (blue line), the 3D-Tunnel (red line), the grey image following the Checkerboard (violet line), and the grey image following the 3D-Tunnel (pink line). The arrows point to the P100, N140, and P220 components.*

As shown in Figure 3B of the study ERP analysis, the discrimination by visual inspection between the 3D-Tunnel and Checkerboard stimuli was more difficult when randomly chosen single trials originating from all subjects were superimposed. When comparing the superimposition of the single trials corresponding to the Tunnel and the Checkerboard (Figure 11 left) with those corresponding to the Tunnel grey screens and the Checkerboard grey screens (Figure 11 right), the visual discrimination of the latter conditions was even more difficult.


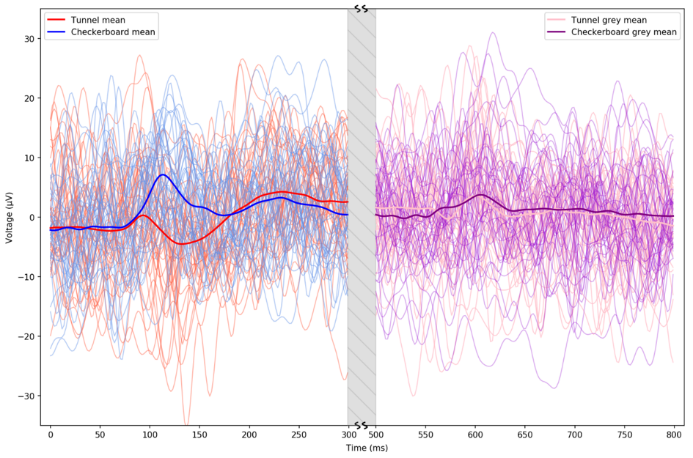


**Figure 11**. *(****Left****)* *Superimposition of EEG signals from single trials recorded between 0 and 300 ms on electrode Oz from* *40 randomly selected trials of Tunnel stimuli and 40 randomly selected trials of Checkerboard stimuli throughout all the subjects* *on which the grand average signals of all trials and all subjects corresponding to the Tunnel stimuli (red lines, n=2880 (192 trials x 15 subjects)) and the Checkerboard (blue lines, n=1440 (96 trials x 15 subjects)) are superimposed.* *(****Right****) The same superimposition of EEG signals from single trials recorded between 500 and 800 ms as in (left) are displayed for the Tunnel greys stimuli (pink lines, n=2880 (192 trials x 15 subjects)) and the Checkerboard greys (violet lines, n=1440 (96 trials x 15 subjects)).*

An electrode selection was also performed with the EEG signals of grey images in order to compare the amount of discriminative information from Tunnel/Checkerboard and grey images with regards to the number and position of the 12 arbitrarily selected electrodes. As a result, Figure 12A illustrates that the classification score for the Tunnel vs. Checkerboard reaches a global maximum using 9 electrodes, while the classification score for Grey Screens reaches its global maximum using all 12 electrodes.

Furthermore, in order to test which scalp region contains the most discriminative information, we used a triad of electrodes corresponding to the occipital, parietal, central, and frontal regions on which the classification pipeline was applied. Figure 12B illustrates this result, including the classification based on grey images. In contrast with the discriminative trend found in the study for the Tunnel vs Checkerboard classification, none was found for the classification of grey images, where the occipital, parietal, central, and frontal zones scored respectively 54%, 56%, 54%, and 57%.


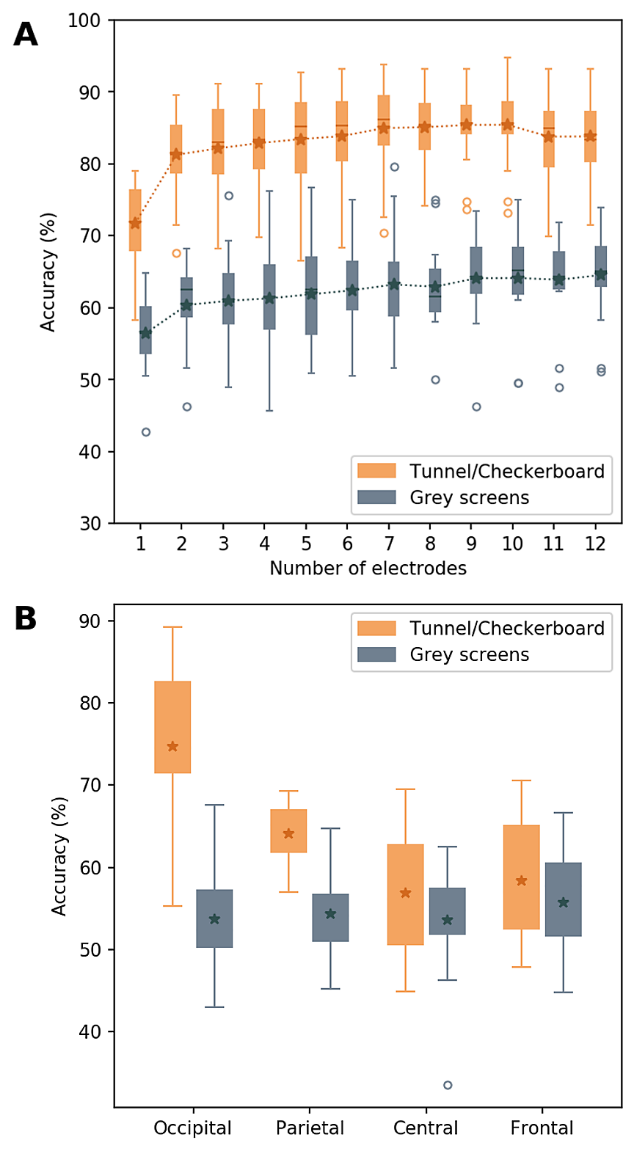


**Figure 12.** *Distribution of the Tunnel vs Checkerboard and Tunnel-Grey vs Checkerboard-Grey inter-subject cross-validated classification accuracy without inverse modeling with respect to the number of electrodes (****A****) and by scalp region (****B****) using only 3 electrodes from each locus (O1, O2, and Oz for the occipital locus, P3, P4, and Pz for the parietal locus, C3, C4, and Cz for the central locus, F3, F4 and Fz for the frontal locus).*

Similarly to the main study, the results of the classifications pipelines were illustrated by the ROC curve (along with the AUC), the Precision-Recall curve (along with the Average Precision), and the confusion matrix. Figure 13 illustrates these performance metrics computed on the results from the inter-subject classification pipelines without inverse modeling. The ROC curve for the Tunnel vs Checkerboard discrimination is more arched than the one for Grey Screens (Figure 13A), which is confirmed by the higher AUC of the first (0.92) compared to the latter (0.7). These results were further validated by the Precision-Recall curves (Figure 13B). Besides, the confusion matrices (Figure 13C) show a balanced recognition accuracy for Tunnels and Checkerboards, although both accuracies are significantly higher for the Tunnel vs Checkerboard (Figure 13C left) than the Grey Screens (Figure 13C right). The inter-subject Tunnel vs Checkerboard without inverse modeling classifier and the inter-subject Grey Screens without inverse modeling classifier reached an accuracy of 84% and 64 % respectively.


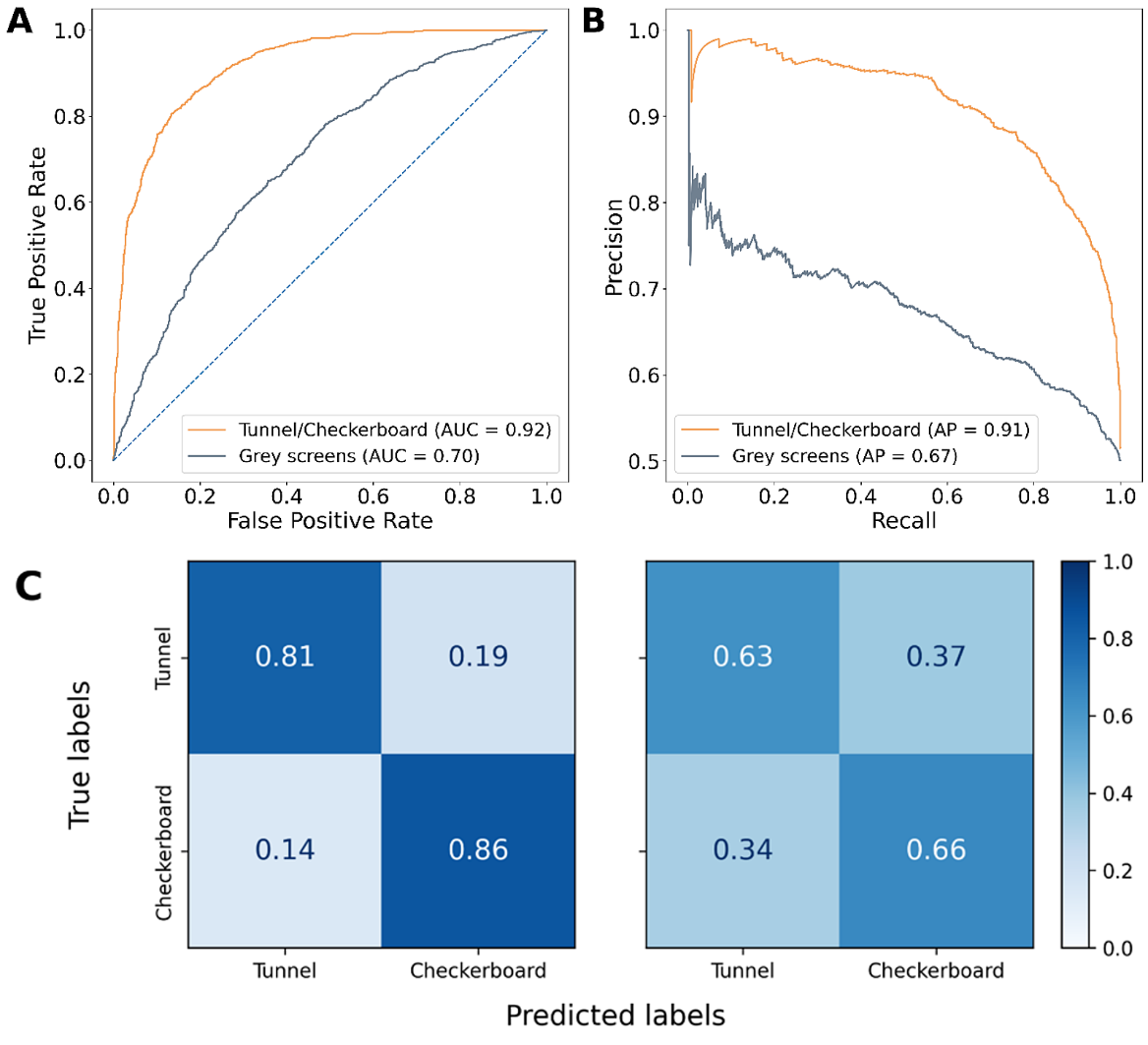


**Figure 13.** *ROC (****A****) and Precision-Recall (****B****) curves for the inter-subject classification of Tunnel vs Checkerboard and Grey Screens without inverse modeling. (****C****) Confusion matrices for the inter-subject classification of Tunnel vs Checkerboard (left) and Grey Screens (right) without inverse modeling.*

Figure 14 illustrates the same performance metrics computed on the results from the intra-subject classification pipelines without inverse modeling. As expected, when trained on the same dataset, the intra-subject Tunnel vs Checkerboard and Grey Screens classification pipelines without inverse modeling reached higher accuracy scores than their inter-subject counterparts. We observed a classification accuracy of 93% and 81% for the discrimination of Tunnel vs Checkerboard and Grey Screens respectively, which corresponds to an increase of 9% and 17% from the inter-subject Tunnel vs Checkerboard and Grey Screens accuracy scores. The ROC curves (Figure 14A), the Precision-Recall curve (Figure 14B), and the confusion matrices (Figure 14C) confirmed the improved accuracy results. The ROC curve (Figure 14A) for the Tunnel vs Checkerboard discrimination task was more arched than that for the Grey Screens, which was confirmed by the higher AUC of the first (0.98) compared to the latter (0.89).


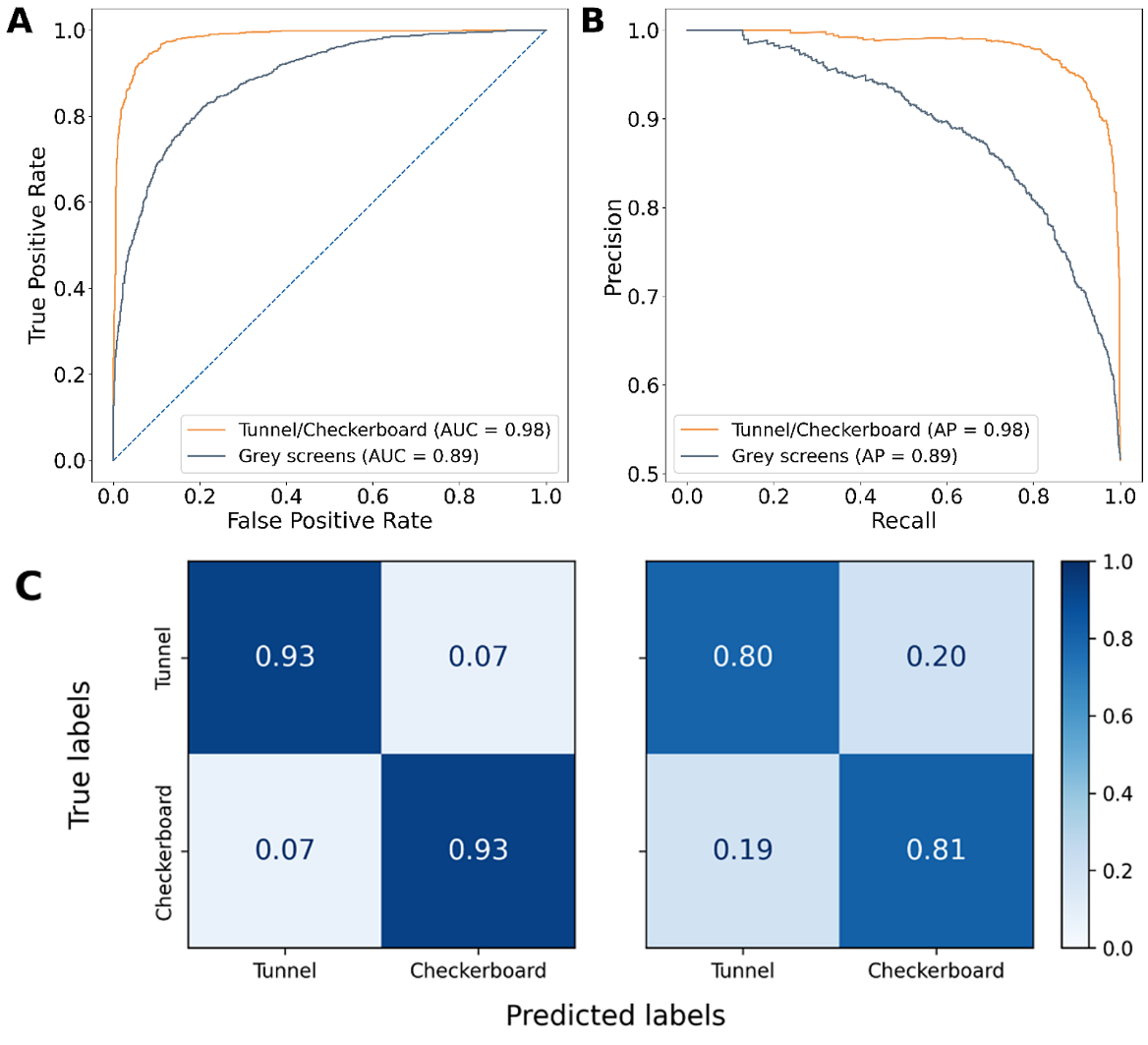


**Figure 14.** *ROC (****A****) and Precision-Recall (****B****) curves for the intra-subject classification of Tunnel vs Checkerboard and Grey Screens without inverse modeling. (****C****) Confusion matrices for the intra-subject classification of Tunnel vs Checkerboard (left) and Grey Screens (right) without inverse modeling.*

The boxplots in Figure 15 summarizes the comparative analysis of the classification pipelines with respect to the intra-subject or inter-subject conditions. The classification performances for the Grey Screens Tunnel vs Checkerboard in the inter-subject and intra-subject conditions were statistically significantly higher than chance (p < 0.001). This latter statistical comparison was computed using a one-sample Wilcoxon test.


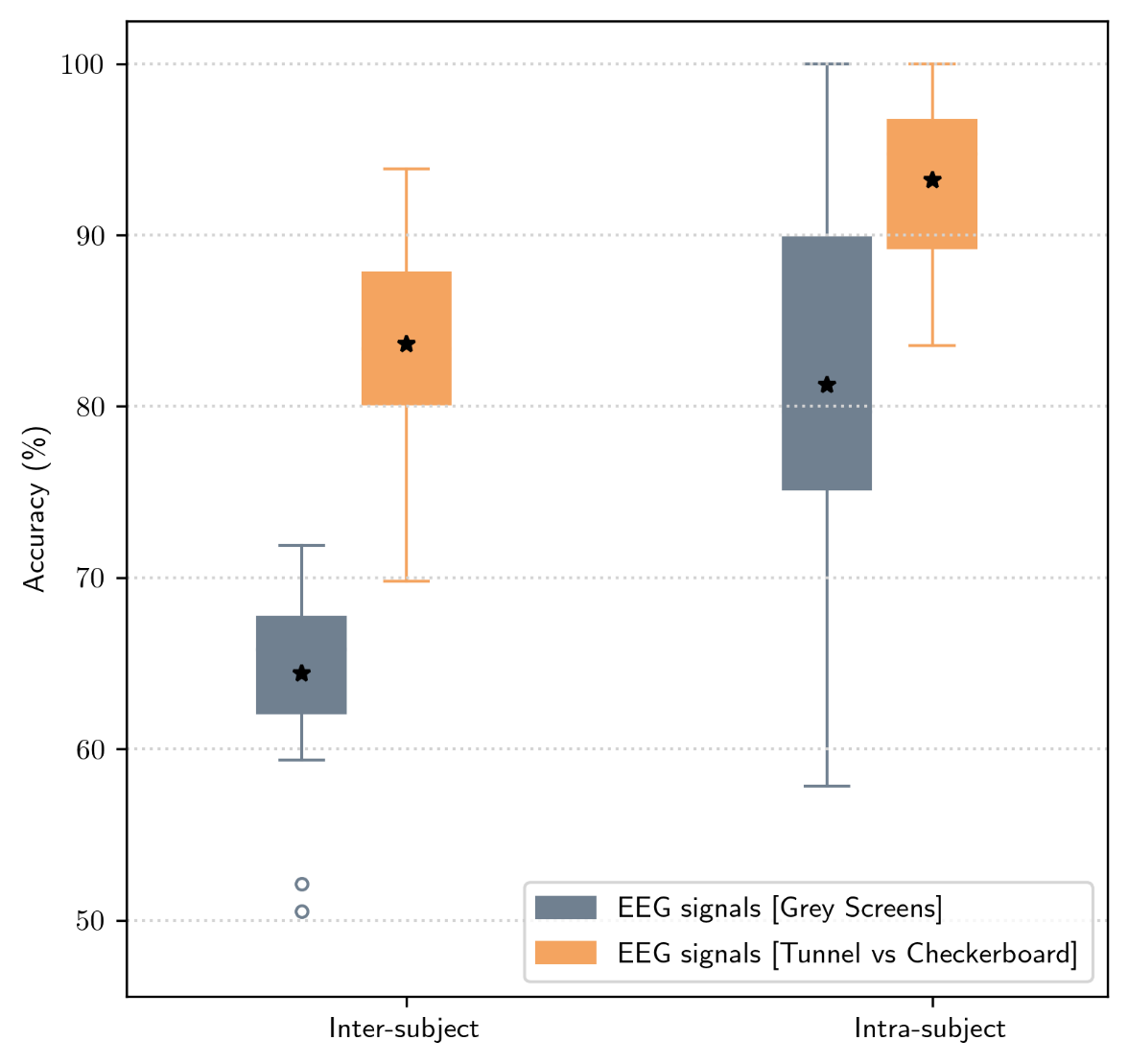


**Figure 15.** *Summary of the performances of the classification pipelines for inter-subject and intra-subject classification of EEG signals from the Tunnel vs Checkerboard (light orange) and Grey Screens (grey) conditions.*

These results, more specifically the fact that our classification pipeline trained with grey images reached performances statistically significantly higher than chance, lead us to acknowledge that the content of grey images remains influenced by, and carries some discriminative information about, the preceding visual stimulus presented to the subject.

**Reference**

[1] C. Simar *et al.*, “Covariance matrices of EEG signals and estimated cortical sources from 15 subjects presented with visual stimuli.” figshare, Jul. 16, 2021. doi: 10.6084/m9.figshare.14997735.v1.
