# Supplementary material for: Riemannian classification of single-trial surface EEG and sources during checkerboard and navigational images in humans
Source: PLoS One. 2022 Jan 14;17(1):e0262417. doi: 10.1371/journal.pone.0262417 (PMC8759639; doi:10.1371/journal.pone.0262417)
Supplement: Attachment — Submitted filename: Review_PONE-D-21-04037_001.pdf [file pone.0262417.s002.pdf]

The Authors Cédric Simar et al. proposed a new approach to classify ERP evoked by different visual stimuli, at single trial level. To do so, they did identify a classification pipeline based on the Riemannian geometry of covariance matrices applied to EEG scalp signals and reconstructed source signals (sLORETA algorithm employed for the purpose). It is certainly not a novel topic considering the wide attention that it has always garnered, especially in the field of BCI applications, but the results reported in the present paper are relevant and promising. Although the proposed methodological framework is in my opinion robust, a more precise description is needed in order to make those results reproducible and, more importantly, applicable to several other applications different from visual stimulation. The impact of this paper would benefit from the possible generalization of the classifier performance in other contexts. Although I do not think that further analyses are necessary, major revisions in the description of the experiment and in the presentation of the results need to be done before publishing the paper. In the present shape, I found it confusing and almost not informative as a consequence. I suggest the Authors stressing the hypothesis behind this work and even more the impact. In the following, I listed more specific comments.

1. In the Introduction section there is a general lack of information and connection between the different parts of this study and it does not provide a comprehensive description of the state of the art able to clearly define the space in which this paper will make a difference. The employment of this method for BCI applications is not even mentioned so it is difficult to understand the importance of moving from the GA approach, used successfully for decades. Another example is the fact that the Authors mentioned the results relative to the classification accuracy for the inter- and intra-subject condition without first introducing those concepts and the reason why both those analysis were performed. Since the objective of the paper is to classify at single trial level, it is not obvious to understand how inter-subjects data should be involved in the training phase and what the hypothesis behind this choice is.
2. Why did the Authors design the two visual tasks with unbalanced number of trials (96 trials for the Checkerboard task and more than double for the 3D-Tunnel)? My guess is that this choice was dictated by the fact that the 3D-Tunnel can be presented

in 4 different orientations and a decent amount of trials is required for each of them in order to test possible differences between them. Please include the rationale behind this choice in the paragraph 2.2.

3. Paragraph 2.3 – The sentence "*Checkerboard pattern and the 3D-Tunnel were alternatively presented with a uniform grey image (Figure 1A, B)*" is confusing in addition to poorly written. The adverb "alternately", for example, is more appropriate than "alternatively". The figure certainly helps but reading this description it is not clear how the stimuli are actually presented. It suggests that *Checkerboard pattern* and *3D-Tunnel* are presented within the same run along with gray screens, which is not correct. Please, rephrase.
4. The paragraph 2.4 is called "EEG data treatment and event related potentials". Please replace event related potentials with ERP for consistency with the rest of the paper. More important is the fact that ERP are the title but not even mentioned in the text. Please, provide some details about the ERP analysis maybe justifying the choice of the windows of interest, which are the ERP the Authors are interested in and they expect to see in each experimental condition with specific latencies. The sentence at the beginning of paragraph 3.1: "As previously observed by our group [7], the P100 component evoked by the Checkerboard was of higher amplitude than the P100 evoked by the 3D Tunnel, which presented a biphasic configuration during the time of the monophasic classical P100 related to the Checkerboard", is a good example and should be moved either in the Introduction or in the Method section.
5. Still in the same paragraph (2.4), the Authors provided a list of the 12 EEG channels used for the ERP analysis and subsequential classification. However, most of the results obtained at scalp level are presented for Oz only. This becomes clear only reading the caption of the reported figures. Please, include this information both in the Method section and in the Result section and provide an explanation for this choice, even simply specifying whether it is because ERP results were similar for all the sensors or, vice versa, because Oz was gave the only meaningful results (mentioning the analysis presented in the Paragraph 3.2.1 that should also be described in the method section instead of the results section directly).

6. Left earlobe electrode as EEG reference is not a popular choice for scalp data acquisition. Please justify.
7. In the description of the source analysis, the Authors did not specify the number of scalp sensors (probably all of them) and reconstructed sources. The fact that only 12 of them are included in the ERP analysis might lead to the wrong conclusion that those signals are also enough to estimate accurately the brain activity in cortical sites. Important aspects of the analysis like the chosen atlas and the number of sources of interest is discussed only in the paragraph 2.8 - which should instead focus on the classification pipeline - making the methodological framework confusing. Other important information, such as whether they considered the reconstructed signal on the centroid of each region of interest or the average of all dipoles, or even the number of voxels used in the lead-field are also missing. The Authors mentioned "cortical regions average" but this step is not clearly described anywhere. Those details are necessary to reproduce the results without using the MNE software chosen by the Authors.
8. The Authors wrote that measures like ROC curves, Precision-Recall curves and confusion matrices are appropriate since the dataset is not balanced (paragraph 2.8). Can they please explain more this relationship? I think that those are the best measures to evaluate the performances of a classifier regardless of the number of trials difference. In this case, that sentence might be inaccurate.
9. I strongly suggest revising Paragraph 3.1, including the captions of Figure 4 and 5. The results description is not clear and very long sentences end up not carrying any message. Here few examples: i) "visual distinction" is a poor choice when the main result here is the distinction between 2 visual stimuli. I would replace that expression with "The discrimination by visual inspection between the 2 stimuli, was not possible anymore if/when…" ii) what's a "single raw EEG signal"? Does it mean single trial? iii) Panels A and B in Figure 5 are redundant. My suggestion is either to report a plot similar to Figure 4 (it would be the most consistent way to present the results besides making immediate the comparison between the two conditions) or to keep only one of the two current panels.
10. Figure 1: please improve the quality/resolution of the 3D-TUNNEL image.

11. Paragraph 3.2.1: the title of this section is not precise because most of the following text refers to the validation of the number of electrodes. I suggest something like: "The effect of the electrodes number and position". I also would appreciate some clarifications: considering that no statistical analysis has been performed and reported in Figure 6A, how would the Authors justify that (for example) the condition 9 electrodes is better than 10? Which qualitative measures did they observe? Also, it is not clear to me how the classifier was trained. Is it "*on the single trials of all subjects*" in both cases? Should this evaluation be performed for inter- and intra-subjects classification separately to be sure that the best number and position of the electrodes is the same?
12. In the same vein of my previous comment, did the Authors considered performing a similar analysis after the source reconstruction? If I understand correctly, the final classifier evaluation without inverse modeling, has been performed using 3 occipital electrodes. How did the Authors use the data from 75 cortical sources? Did they consider their optimal number and location? If not, why did they non consider it an interesting aspect of the analysis? Reducing the number of ROIs might be a key aspect of this pipeline and it would reduce the computational time. Something about this topic has been done by the Authors and reported in the second half of Paragraph 3.2.4 but I could not understand in which way these results are related with the classification analysis and if they are just a mere observation of the brain activations. I want to conclude this comment saying that the importance and relevance of the presented results are completely hidden by the lack of details (both in the main text and in the figures caption), by the quality of the text and by the poor organization of the paragraphs. I strongly suggest the Authors to focus on improving the presentation of the results.
13. Figure 7/8/9: For the sake of clarity and consistency between the results obtained before and after the source reconstruction, I suggest reporting figures containing the same information. If the comparison between inter- and intra-subjects coded by red and pink lines respectively have been chosen for the classification with inverse modeling, similar figures should be reported for the classification without inverse modeling. I suggest combining figure 7 and 8 removing the results relative to the gray

screen (eventually the Authors can report them separately in the supplementary). The alternative would be to report the results for the gray screens classification obtained in the source space.

14. The discussion section needs to be improved on the same aspects I listed so far for the other sections with an additional lack of continuity between the different parts. The discussion opens with several comments very specifically BCI-related when this concept was barely touched in the introduction and in the abstract. I suggest i) revising the text because not well written and difficult to follow; ii) mention that contributing to improve the use of ERP in BCI is one of the main goals of the paper toward the end of the Introduction. As I pointed out several times, there is a general lack of common thread throughout the whole paper and highlighting the purpose and which results play the most important role in achieving this goal might help the reader to orient among the different sections.
15. The Authors should acknowledge somewhere the limitation of the study. They can decide to dedicate a separate paragraph of the paper or to add some comments at the end of the discussion. The main points I suggest mentioning are the limited number of subjects (particularly meaningful because we are talking about healthy subjects performing an easy task and because they are used to validate a new approach for the first time) and the fact that the results need to be taken with caution because only tested on two visual stimuli under extremely controlled conditions (no peripheral view). The Authors do not have to forget the final purpose of the classification at single trial level, which is the ability to use measures like ERP in real time in neurofeedback or BCI applications, particularly meaningful in the rehabilitation field where the signals might be acquired in less ideal conditions without significantly degrading the performance of the classifier. If the Authors are thinking making available the code for their pipeline, this should be mentioned because more scientists could apply it and help validating/improving the results obtained on these 15 subjects.
16. Please double check for typos and inaccuracies. Here few examples:

a. The introduction opens with a confusing sentence in which the words “evoked potentials” appear twice. I suggest deleting “which generate the well-defined components of the evoked potentials” that might be redundant.
b. Introduction: “In THE PRESENT STUDY, we use a classification pipeline based on xDAWN spatial filtering [23] and Riemannian geometry applied TO SINGLE-TRIAL EEG DATA recorded during A visual stimulation”.
c. Figure 8C: please add a label to specify that the confusion matrix on the left refers to the actual visual stimulus and the one on the left to the gray screens. Also, as a general comment, the confusion matrices are too big considering that the only relevant information is the number in the middle of each square.
d. Figure 9: The Authors never talked about “patients”. Please replace with “subjects”.
e. I suggest replacing everywhere “Raw data” with “Scalp data” or “Sensors data”. EEG data filtered and segmented are not “raw”.
