# Supplementary material for: Riemannian classification of single-trial surface EEG and sources during checkerboard and navigational images in humans
Source: PLoS One. 2022 Jan 14;17(1):e0262417. doi: 10.1371/journal.pone.0262417 (PMC8759639; doi:10.1371/journal.pone.0262417)
Supplement: Attachment — Submitted filename: Response to Reviewers.pdf [file pone.0262417.s004.pdf]

Reviewer #1:

Reviewer's comment:

Thank for making the material available as per Plos policy. As for the manuscript, and given the reviewer 2 revision I think it now look great and easy to follow. Yet, I have a major problem with the manuscript. The paper shows, convincingly, that XDAWN+Riemannian projection at the geometric mean gives good results. The problem is that other classification pipelines not using XDAWN and not using Riemannian geometry can also discriminate between conditions. In my opinion, for a publication in a general outlet you need more than showing it works, you need a comparison. Not a comparison to a grand average, which make no sense since the framework (fitting vs predicting) are different, but a comparison of performances with the data without XDAWN or without projecting onto the tangent space. My point is you need to show it outperforms other approaches or at least provides a more straightforward interpretation.

Given the authors track record, I'm sure they are well aware of the various other methods out there. Just pointing out for instance in the introduction when it sounds like there is noting else but grand averages and single trial analyses are new ... well see eg https://www.frontiersin.org/articles/10.3389/fpsyg.2011.00322/full and references therein - starting with Donchin, E. (1969). Discriminant analysis in average evoked response studies: the study of single-trial data. Electroencephalogr. Clin. Neurophysiol. 27, 311–314.

Concretely, my suggestion is to a logistic regression on the data, then with XDAWN, then in tangent space without DAWN - that way we have all 4 ways to process and show your approach works best ; otherwise you just say that you can classify like many other can. I don't mean to sound harsh, I think the method is great and the paper is well written, but it's not convincing enough, especially in a generic outlet.

Authors' response:

Thank you for your important comment and suggestion. In the previous version of the manuscript, although we showed that our approach (xDAWN filtering and Riemannian projection) is able to discriminate between the presentation of the two displays, we did not indeed show a comparison of multiple pipelines and thus did not show that our approach outperformed other approaches.

You are right that it is important to show the comparative advantage of our method with respect to other classification pipelines and this would be a considerable improvement of the manuscript, as well as important information for the readers. Therefore, in the new version of the manuscript, we added section "3.2.5 Comparison of classification pipelines" that describes and compares 5 additional classification pipelines with our approach in order to show the benefits of applying xDAWN filtering and Riemannian projection, as well as to establish the robustness and generalizability of our approach with respect to the other classification pipelines. As you suggested, the additional pipelines include the use of a logistic regression on the vectorized EEG signals or cortical sources, as well as the selective use of xDAWN filtering and Riemannian projection. We also added a classification pipeline using the CSP algorithm. The results of these comparisons are presented with statistical significance tests computed with a Mann–Whitney U test.

These results show that

- the classification pipeline used in our approach outperforms the other pipelines in both intra-subject and inter-subject conditions, using either EEG signals or cortical sources
- xDAWN spatial filtering significantly improves inter-subject generalizability
- the projection onto the tangent space significantly improves the classification accuracy in the intra-subject condition
- the classification pipeline used in our approach significantly outperforms the CSP algorithm in the inter-subject condition

Please find hereunder the complete section that we added in the new version of the manuscript.

Lastly, the reference you suggested, referring to the relevance of single-trial analysis for neuroimaging data, was interesting and we thus included this sentence in the new version of the manuscript: "The relevance of considering single-trial analyses with neuroimaging data is also discussed in [42]."

3.2.5 Comparison of classification pipelines

As suggested by a reviewer, after the comparative analysis in section 2.8, we compare here the classification accuracy of our approach with other classification pipelines using the same cross-validation methodology. The purposes of this comparison are to (i) quantify the benefits of applying xDAWN filtering and projecting covariance matrices onto the tangent space, and (ii) establish the robustness and generalizability of our approach with respect to other classification pipelines.

To that end, Figure 9 compares the mean cross-validated accuracy, with respect to the intra-subject or inter-subject conditions, computed on the following classification pipelines with and without inverse modelling:

- *LR on vectorized EEG signals/sources activations*: the EEG signals or cortical sources activations are unrolled into a 1-dimensional vector and classified using a logistic regression (LR).
- *Covariance matrices and LR*: covariance matrices of EEG signals or cortical sources are estimated, unrolled into a 1-dimensional vector and classified using a logistic regression.
- *Covariance matrices, TS and LR*: covariance matrices of EEG signals or cortical sources are estimated, projected onto the tangent space and classified using a logistic regression.
- *xDAWN covariance and LR*: covariance matrices are estimated from the result of xDAWN spatial filtering applied on EEG signals or cortical sources, unrolled into a 1-dimensional vector and classified using a logistic regression.
- *CSP and LR*: the output of the Common Spatial Patterns (CSP) algorithm [59], [60] applied on EEG signals or cortical sources is classified using a logistic regression.

- *xDAWN covariance, TS and LR*: covariance matrices are estimated from the result of xDAWN spatial filtering applied on EEG signals or cortical sources, projected onto the tangent space and classified using a logistic regression.

The comparison of classification results from these different pipelines illustrated in Figure 9 highlights several interesting patterns. Firstly, we can observe that the classification pipeline used in our approach outperforms the other pipelines in both intra-subject and inter-subject conditions, using either EEG signals or cortical sources. Secondly, we observe that the use of xDAWN spatial filtering significantly improves the classification accuracy in the inter-subject condition ($p < 0.001$), i.e. inter-subject generalizability, but not in the intra-subject condition ($p > 0.001$). Whereas, conversely, the use of the projection onto the tangent space significantly improves the classification accuracy in the intra-subject condition ($p < 0.001$) but not in the inter-subject condition ($p > 0.001$), although this latter absence of accuracy improvement in the inter-subject condition may also be explained by the difficulty to discriminate inter-subject EEG signals and cortical sources without xDAWN spatial filtering for this task. Lastly, we observe that the classification pipeline used in our approach significantly outperforms the CSP algorithm in the inter-subject condition ($p < 0.001$) but not in the intra-subject condition ($p > 0.001$). These statistical comparisons were computed using a Mann–Whitney U test.

**Classification pipeline**	**Mean accuracy**	
**EEG signals**	**Intra-subject**	**Inter-subject**
LR on vectorized EEG signals	0,49	0,67
Covariance matrices and LR	0,47	0,51
Covariance matrices, TS and LR	0,89	0,55
xDAWN covariance and LR	0,48	0,52
CSP and LR	0,86	0,56
xDAWN covariance, TS and LR *	**0,93**	**0,84**
**Cortical sources**		
LR on vectorized sources activations	0,57	0,58
Covariance matrices and LR	0,66	0,56
Covariance matrices, TS and LR	0,81	0,53
xDAWN covariance and LR	0,53	0,57
CSP and LR	0,77	0,51
xDAWN covariance, TS and LR *	**0,82**	**0,63**

Figure 9. *Comparison of the mean accuracies reached by different inter-subject and intra-subject classification pipelines based on EEG signals and (sub)cortical sources. The asterisk designates the classification pipeline used in our approach.*

Reviewer #3:

Reviewer's comment:

All comments on the manuscript have been reviewed. The manuscript has been considerably improved and is ready for publication in my opinion.

Authors' response:

Thank you for your previous comments that contributed to the considerable improvement of the manuscript and for accepting it for publication.
